# Pooled cohort study on height and risk of cancer and cancer death

**DOI:** 10.1007/s10552-013-0317-7

**Published:** 2013-10-31

**Authors:** Sara Wirén, Christel Häggström, Hanno Ulmer, Jonas Manjer, Tone Bjørge, Gabriele Nagel, Dorthe Johansen, Göran Hallmans, Anders Engeland, Hans Concin, Håkan Jonsson, Randi Selmer, Steinar Tretli, Tanja Stocks, Pär Stattin

**Affiliations:** 1 Department of Surgery and Perioperative Sciences, Urology and Andrology, Umeå University, 901 87 Umeå, Sweden; 2 Department of Medical Statistics, Informatics and Health Economics, Innsbruck Medical University, Innsbruck, Austria; 3 Department of Plastic Surgery, Skåne University Hospital Malmö, Lund University, Malmö, Sweden; 4 Department of Global Public Health and Primary Care, University of Bergen, Bergen, Norway; 5 Norwegian Institute of Public Health, Oslo/Bergen, Norway; 6 Institute of Epidemiology and Medical Biometry, Ulm University, Ulm, Germany; 7 Agency for Preventive and Social Medicine, Bregenz, Austria; 8 Department of Surgery, Skåne University Hospital Lund, Lund, Sweden; 9 Department of Public Health and Clinical Medicine, Nutritional Research, Umeå University, Umeå, Sweden; 10 Department of Radiation Sciences, Oncology, Umeå University, Umeå, Sweden; 11 Institute of Population-based Cancer Research, Cancer Registry of Norway, Oslo, Norway

**Keywords:** Body stature, Body height, Epidemiology, Cancer risk, Cohort study

## Abstract

**Purpose:**

To assess the association between height and risk of cancer and cancer death.

**Methods:**

The metabolic syndrome and cancer project is a prospective pooled cohort study of 585,928 participants from seven cohorts in Austria, Norway, and Sweden. Hazard ratios (HRs) and 95 % confidence intervals (CIs) for cancer incidence and death were estimated in height categories and per 5-cm increment for each cancer site using Cox proportional hazards model.

**Results:**

During a mean follow-up of 12.7 years (SD = 7.2), 38,862 participants were diagnosed with cancer and 13,547 participants died of cancer. Increased height (per 5-cm increment) was associated with an increased overall cancer risk in women, HR 1.07 (95 % CI 1.06–1.09), and in men, HR 1.04 (95 % CI 1.03–1.06). The highest HR was seen for malignant melanoma in women, HR 1.17 (95 % CI 1.11–1.24), and in men HR 1.12 (95 % CI 1.08–1.19). Height was also associated with increased risk of cancer death in women, HR 1.03 (95 % CI 1.01–1.16), and in men, HR 1.03 (95 % CI 1.01–1.05). The highest HR was observed for breast cancer death in postmenopausal women (>60 years), HR 1.10 (95 % CI 1.00–1.21), and death from renal cell carcinoma in men, HR 1.18 (95 % CI 1.07–1.30). All these associations were independent of body mass index.

**Conclusion:**

Height was associated with risk of cancer and cancer death indicating that factors related to height such as hormonal and genetic factors stimulate both cancer development and progression.

## Introduction

Adult height, determined by genetics and by nutrition in childhood, has been associated with an increased risk of some cancers such as cancer of the prostate [1], breast [2], colorectum [3], ovary [4, 5], pancreas [6], kidney [7], testis [8], endometrium [9], malignant melanoma [2, 10, 11], and with lymphohematopoietic malignancies [12]. However, there are only a few studies on all cancer sites, [2, 13–15] and only one of these studies included men as well as women [13]. Height has also been associated with an increased risk of cancer death in contrast to the *decreased* risk of total mortality and mortality from cardiovascular diseases [16, 17]. To the best of our knowledge, no large study to date has analyzed risk of cancer at all sites and cancer death in the same study.

The aim of this prospective cohort study was to assess the association between height and risk of cancer and cancer death in a large prospective cohort in order to provide precise estimates for risk of incident cancer and cancer death overall and for specific cancer sites.

## Materials and methods

### Study population

This study was conducted within the metabolic syndrome and cancer project (Me-Can), which consists of data from health examinations performed in seven cohorts, which have been described in detail previously [18]. In brief, the Me-Can project includes cohorts from Norway; the Oslo study I cohort (Oslo), Norwegian Counties Study (NCS), Cohort of Norway (CONOR) and Age 40-programme (40-y), from Sweden; Västerbotten Intervention Project (VIP) and Malmö Preventive Project (MPP) from Austria; Vorarlberg Health Monitoring and Prevention Program (VHM&PP).

As part of the health examination data on height, weight and smoking status were obtained. In all cohorts, weight and height were measured with participants wearing light indoor clothes and no shoes, and height was measured to the nearest centimeter.

We only used data from first health examination [18], and we excluded participants with height below 100 or above 250 cm (1 participant), and participants with missing value for height (3,412 participants). To account for age-induced shrinkage, we further excluded participants with health examination at age 80 years or above (4,551 participants). Due to policy restrictions imposed by the Norweigan Institute of Public Health that the proportion of Norweigan participants in Me-Can studies could not exceed 50 %, we randomly excluded participants from the Norweigan sub-cohorts to the final dataset. The Me-Can project was approved by research ethics review boards in the respective countries.

### End points

Cancer diagnoses were identified through linkages with the National Cancer Registry in Sweden and Norway and Vorarlberg State Cancer Registry in Austria [19–21]. The International Classification of Diseases, seventh revision (ICD-7) was used for identification of cancer cases. In Norway and Sweden, data were also linked to the Registry of Total Population and Population Changes for assessment of vital status (data not available in Austria). Causes of death were coded according to Eurostat European shortlist for causes of death [22] and were obtained by linkage to National Cause of Death Registry in each country.

### Statistical methods

Hazard ratios and 95 % confidence intervals (95 % CI) for increased height were analyzed with Cox proportional hazards regression with attained age as the time scale. Participants were followed from date of health examination until date of cancer diagnosis or death of cancer, or until censoring at the date of death from any cause, emigration, or end of follow-up (for analysis of cancer: 31 December 2003 in Austria, 2005 in Norway, and 2006 in Sweden; for cancer death: 31 December 2003 in Austria, and 2004 in Norway and Sweden), whichever occurred first.

The Cox models were adjusted for ten categories of date of birth and ten categories of age at health examination, and stratified for sub-cohort within the model. The proportional hazards assumption was tested using Schoenfeld residuals and found valid for this model. For total cancer and cancer death, we calculated hazard ratios (HRs) in categories of height. For total cancer and cancer death and also for specific sites, we calculated HRs using height as a continuos variable for 5 cm increment in height.

### Effect modification by BMI, smoking and birth cohort

Body mass index (BMI; weight/height^2^ (kg/m^2^)) was divided into categories as defined by WHO (<25, 25 to <30, 30 kg/m^2^ and above) [23], and smoking was classified as never-smoker, ex-smoker, and smoker. We tested for multiplicative interaction between categories of BMI or smoking, and 5 cm increment in height, using likelihood ratio test for total cancer and for each site. Since we tested for interaction for each site, around 20 tests of multiplicative interactions were performed for each sex and each exposure, and thus, we adjusted the significance level for multiple testing using the Holm–Bonferroni correction [24]. For those cancer sites where interaction was found, we further investigated this by calculating HRs separately for each strata of BMI or smoking.

We specifically examined potential effect modification using a separate model with only subjects from Austria and Norway born before 31 December 1945, since participants in these countries may have been affected by dietary restrictions [25]. Similar to above, the significance level was adjusted with the Holm–Bonferroni correction.

### Menopause

To investigate whether the association with height differed by menopausal status, we calculated HRs of breast cancer in groups according to age at diagnosis; assuming that women before age 50 had not undergone menopause (premenopausal), women aged 50–60 were equivocal regarding menopausal status (perimenopausal) and that women above age 60 years had undergone menopause (postmenopausal).

### Smoking-related cancers

Smoking-related cancers were defined according to definition by International Agency for Research on Cancer and include cancer of the cervix, colorectum, kidney, liver/intrahepatic bile ducts, larynx/trachea/bronchus/lung, lip/oral cavity/pharynx, esophagus, pancreas, stomach, bladder, and bone marrow (acute and chronic myeloid leukemia) [26]. All remaining cancers except those in the category “Other cancers” were defined as not smoking-related cancers. We calculated HRs for height in current and never smokers in not smoking-related and smoking-related cancers.

All statistical tests were two-sided, and *p* values lower than 0.05 were considered statistically significant. Calculations were performed with STATA MP/2 version 11.2.

## Results

The pooled study cohort consisted of 297,156 women and 288,772 men, in total 585,928 participants with a mean age at health examination of 43.1 years (SD = 11.0), and mean year of birth 1949 for women and 1948 for men. Mean follow-up time was 12.7 years (SD = 7.2) and mean height at health examination was 164.3 cm (SD = 6.3) in women and 177.3 cm (SD = 6.9) in men, Table 1. During follow-up, 17,549 women and 21,313 men were diagnosed with cancer and 5,431 women and 8,116 men died of cancer. Out of the 6,161 women diagnosed with breast cancer, 1,855 were diagnosed before age 50 (premenopausal), 2,173 were diagnosed between age 50 and 60 (perimenopausal), and 2,133 were diagnosed after age 60 (postmenopausal). Out of the 1,014 women that died of breast cancer, 432 were diagnosed before age 50, 253 were diagnosed between age 50 and 60, and 329 were diagnosed after age 60. Data on BMI and smoking were complete for over 99 % of participants.

**Table 1:** Characteristics of the Me-Can cohort

Sub-cohort	Year of health measurement	% males	Number of subjects	Current smokers (%)		Age at measurement (years)	Follow-up (years)	Cancer cases		Death of cancer		Height (cm)	
				Women	Men	Mean (SD)	Mean (SD)	Women	Men	Women	Men	Women, mean (SD)	Men, mean (SD)
Total		49	585,928	28	33	43.1 (11.0)	12.7 (7.2)	17,549	21,313	5,431	8,116	164.3 (6.3)	177.3 (6.9)
Norway													
Oslo	(1972–73)	100	8,249		56	43.9 (5.7)	26.7 (8.5)		2,061		1,083		177.6 (6.5)
NCS	(1974–88)	50	41,384	40	52	39.8 (7.3)	24.2 (7.1)	2,525	2,706	1,036	1,243	163.3 (6.1)	176.1 (6.7)
CONOR	(1994–2003)	47	60,054	31	31	46.7 (14.4)	7.2 (4.1)	1,271	1,325	440	505	164.4 (6.5)	177.6 (7.0)
40-y	(1985–99)	48	184,469	33	34	42.2 (4.8)	12.1 (4.5)	5,068	3,305	1,259	1,120	165.7 (5.9)	178.9 (6.5)
Austria													
VHM&PP	(1985–2005)	46	173,876	21	29	41.4 (14.9)	11.4 (5.7)	4,629	5,116	1,530	1,897	163.1 (6.5)	175.2 (6.9)
Sweden													
VIP	(1985–2005)	49	84,573	21	18	46.7 (9.7)	9.5 (5.4)	2,125	2,076	543	504	164.5 (6.1)	178.1 (6.7)
MPP	(1974–92)	67	33,323	46	49	45.7 (7.4)	22.0 (7.8)	1,931	4,724	623	1,764	163.7 (6.1)	177.0 (6.8)

Study participants with short stature had a higher BMI than taller participants; 16 % of women below 160 cm were obese (BMI > 30 kg/m^2^) versus 7 % of women above 175 cm, and 12 % of men below 170 cm were obese versus 9 % of men above 185 cm, Table 2. There was a somewhat higher proportion of smokers among tall women: 29 % of women above 175 cm versus 25 % of women below 160 cm. In men, the opposite pattern was seen: 30 % of men above 185 cm versus 35 % of men below 170 cm.

**Table 2:** Distribution of overweight and smoking in height categories in the Me-Can cohort

	BMI			Smoking		
	Below 25*	25 to <30	30 and above	Never-smoker	Former smoker	Current smoker
Women	N (%)	N (%)	N (%)	N (%)	N (%)	N (%)
175 cm and above	10,540 (71)	3,342 (22)	1,035 (7)	6,955 (47)	3,569 (24)	4,329 (29)
170 to <175 cm	32,119 (69)	10,680 (23)	3,481 (8)	22,065 (48)	10,547 (23)	13,461 (29)
165 to <170 cm	55,799 (65)	21,582 (25)	7,843 (9)	42,247 (50)	18,082 (21)	24,459 (29)
160 to <165 cm	52,757 (61)	24,428 (28)	9,899 (11)	45,943 (53)	16,134 (19)	24,511 (28)
Below 160 cm	33,380 (53)	19,964 (31)	10,197 (16)	38,286 (60)	9,062 (14)	15,773 (25)
Men						
185 cm and above	21,243 (51)	16,574 (40)	3,591 (9)	17,583 (42)	11,051 (27)	12,522 (30)
180 to <185 cm	32,646 (49)	28,445 (42)	5,902 (9)	27,623 (41)	17,351 (26)	21,627 (32)
175 to <180 cm	39,561 (48)	34,940 (43)	7,684 (9)	32,953 (40)	20,738 (25)	28,014 (34)
170 to <175 cm	28,032 (45)	27,777 (45)	6,536 (10)	25,562 (41)	14,684 (24)	21,741 (35)
Below 170 cm	15,481 (43)	15,978 (45)	4,305 (12)	15,268 (43)	7,841 (22)	12,467 (35)

* BMI in WHO categories

### Risk of cancer and cancer death in height categories

**Fig. 1:**
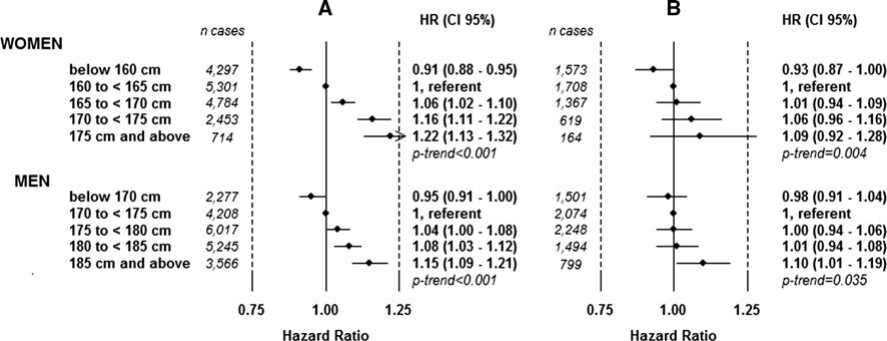
Risk of total cancer (**a**) and cancer death (**b**) by height category for women and men. HRs are adjusted for date of birth and age at health examination, and stratified for sub-cohort within the model

Increasing height was associated with an increased risk of cancer in both women and men: HR 1.22 (95 % CI 1.13–1.22) for women of 175 cm and above compared to women of 160–165 cm, *p*-trend < 0.001, and HR 1.15 (95 % CI 1.09–1.15) for men of 185 cm and above compared to men of 170–175 cm, *p*-trend < 0.001, Fig. 1a. Increasing height was associated with an increased risk of cancer death in women and men: HR 1.09 (95 % CI 0.92–1.28) for women of 175 cm and above compared to women of 160–165 cm, *p*-trend = 0.004, and HR 1.10 (95 % CI 1.01–1.19) for men of 185 cm and above compared to men of 170–175 cm, *p*-trend = 0.0035, Fig. 1b.

### Specific cancer sites

**Fig. 2:**
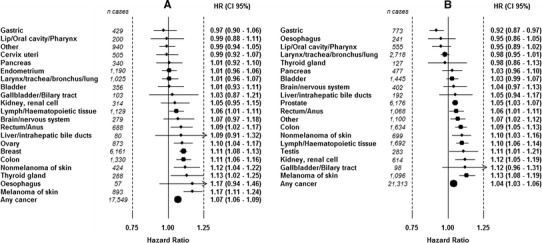
Risk of cancer by 5 cm increment in height for women (**a**) and men (**b**). HRs are adjusted for date of birth and age at health examination, and stratified for sub-cohort within the model

Increasing height (per 5 cm) was associated with an increased risk for total cancer in women, HR 1.07 (95 % CI 1.06–1.09), and in men, HR 1.04 (95 % CI 1.03–1.06), as well as an increased risk for 8 out of 21 cancer sites in women and 9 out of 19 cancer sites in men, Fig. 2a, b. In both sexes, the highest HR was seen for malignant melanoma; in women, HR 1.17 (95 % CI 1.11–1.24), and in men, HR 1.12 (95 % CI 1.08–1.19). Height was not associated with a decreased risk of any cancer in women but was associated with decreased risk of gastric cancer in men, HR 0.92 (95 % CI 0.87–0.97).

### Death from cancer at specific sites

**Fig. 3:**
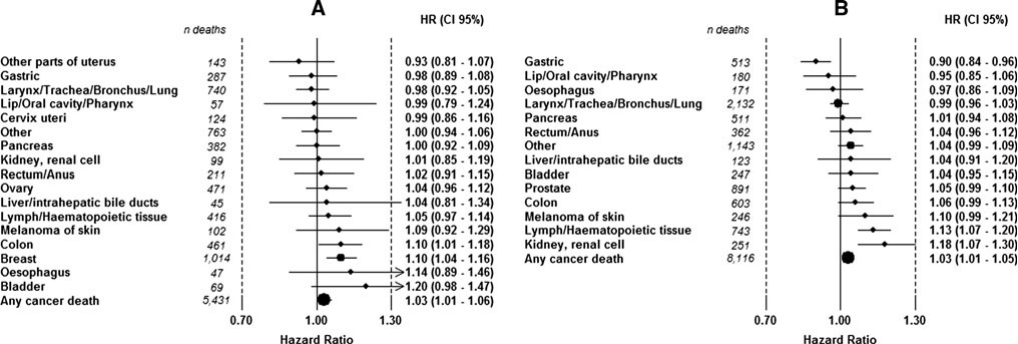
Risk of cancer death by 5 cm increment in height for women (**a**), and men (**b**). HRs are adjusted for date of birth, age at health examination and stratified for sub-cohort within the model

Increasing height (per 5 cm) was associated with an increased risk for total cancer death in women, HR 1.03 (95 % CI 1.01–1.06), and in men, HR 1.03 (95 % CI 1.01–1.05), as well as an increased risk of death from 2 out of 17 cancer sites in women and 2 out of 14 cancer sites in men, Fig. 3a, b. In women, the highest HR was seen for death of breast cancer, HR 1.10 (95 % CI 1.04–1.16), and in men the highest HR was seen for death of renal cell carcinoma, HR 1.18 (95 % CI 1.17–1.30). Height was not associated with a decreased risk of death from any cancer in women but a decreased risk of dying of gastric cancer in men, HR 0.92 (95 % CI 0.87–0.97).

After adjusting the *p* values for multiple testing, we found an interaction between BMI and height for total cancer in men (*p* < 0.001), but no interaction between height and smoking. In subsequent analysis of categories of BMI, HRs for total cancer (per 5 cm increment) was slightly higher in men that were overweight or obese; HR 1.06 (95 % CI 1.05–1.08) for BMI 25–30 and HR 1.07 (95 % CI 1.03–1.11) for BMI > 30, as compared to HR 1.02 (95 % CI 1.01–1.04) for BMI < 25. We found no effect modification among those subjects born before 31 December 1945 in Austria and Norway that may have been affected by nutritional restrictions during World War II, after adjustment for multiple testing.

There was an association between height and risk of breast cancer in postmenopausal women (age > 60), HRs (per 5 cm increment) was 1.11 (95 % CI 1.07–1.15) for incident breast cancer and HR 1.10 (95 % CI 1.00–1.21) for breast cancer death. There were no associations with breast cancer in premenopausal women (age < 50); HR 0.98 (95 % CI 0.94–1.02), or breast cancer death, HR 1.02 (95 % CI 0.94–1.11).

**Fig. 4:**
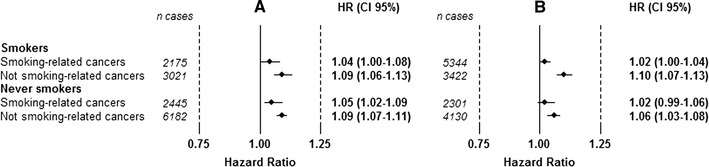
Risk of cancer by 5 cm increment in height for smoking-related and non-smoking-related cancers, in smokers and never smokers, for women (**a**) and men (**b**). HRs are adjusted for date of birth and age at health examination, and stratified for sub-cohort within the model

There were no significant differences in height-associated HRs for total cancer in non-smoking-related cancers and smoking-related cancers in women, Fig. 4. The height-associated HR for total cancer was higher in non-smoking-related cancers than in smoking-related cancers in male smokers; HR 1.10 (95 % CI 1.07–1.13) versus HR 1.02 (95 % CI 1.00–1.04).

## Discussion

In this population-based study with more than 38,800 cancer cases and 13,500 cancer deaths, height was associated with an increased risk of cancer incidence and death for all sites combined. The highest height-related HR for cancer incidence was found for malignant melanoma in both women and men. For shared-site cancers, height was positively associated with risk in both women and men for lymphohematopoietic malignancies, colon and rectal cancer, and non-melanoma and melanoma skin cancer. For thyroid cancer, the association was restricted to women and for renal cell carcinoma to men. The highest HR for cancer death was observed for breast cancer in postmenopausal women and renal cell carcinoma in men. For shared-site cancers, height was positively associated with death of colon cancer in women, and for men the association was of border line significance. Associations to death of lymphohemapoietic malignancies and renal cell carcinoma were restricted to men.

Our study has several strengths, it was large and population-based, it included both cancer diagnosis and death from different cancers, height was measured at health examination, and the end points of cancer diagnoses were obtained through linkages with national cancer registers with almost complete capture of cancer cases [19–21]. However, we did not have information on potential confounders for female cancers, i.e., reproductive factors. Also, we did not have information on socioeconomic status, which might affect both cancer incidence and survival [27]. However, in previous large studies, adjustment for these factors did not affect risk estimates for the association between height and cancer [2, 13, 14].

In our study, as well as in four previous large studies on height and risk of all-sites cancer, height has showed positive association to risk of cancers of the colon, breast, and ovary in women [2, 13–15]. Our finding of positive associations for thyroid cancer and malignant melanoma in women has been reported in three out of these four studies. For men, our finding of positive associations for cancers of the colon, rectum, and prostate are consistent with the only large previous study on all-sites cancer that included both women and men, a Korean cohort study of 9,400 female and 23,700 male cancer cases [13]. Our finding of positive associations for kidney and testicular cancer, and malignant melanoma, are not consistent with the Korean study but are in accordance with several large studies on height and separate cancer sites [7, 8, 11].

In our study, height-associated HR for total cancer was higher in not smoking-related cancers than in smoking-related cancers in male smokers, similar to the finding in women in the Million Women Study, a population-based study including over 97,300 cancer cases [2]. For women in our study, the risk estimates pointed in the same direction as in men, but they were not significant, possibly due to limited number of cancer cases among female smokers.

For cancer death, our findings of positive associations between height and risk of death of breast and colon cancer in women and lymphohematopoietic malignancies and renal cell carcinoma in men are in line with a study on height and the risk of cause-specific death that included over one million individuals and over 47,000 cancer deaths [16], with the exception of renal cell carcinoma which was not included in this study. There has, to our knowledge, been no large study on height and risk of death of kidney cancer or renal cell carcinoma.

Our finding of an inverse association for height and risk of diagnosis and death of gastric cancer in men was not seen in the Korean all-sites cancer study which included 8,777 male cases of gastric cancer [13] but is in accordance with the above-mentioned study on height and risk of cause-specific death which included over 2,040 cases of gastric cancer [16]. Although the study on mortality did not analyze men and women separately but instead adjusted for sex [16], this result is still likely representative for men since the incidence of gastric cancer is twice as high in men as in women [28]. The most important risk factor for gastric cancer is infection with Helicobacter pylori (H. pylori) [28], and as such exposure has been associated with poor growth in children [29], this infection might explain the inverse association between height and gastric cancer seen in our study.

In our study, the highest HRs for height and risk of cancer was for malignant melanoma in both women and men. For women, two other studies on all-sites cancer have also shown highest relative risks for malignant melanoma [2, 14], while the all-sites cancer study that included both women and men did not report results for malignant melanoma separately from other skin cancers [13]. In the present study, height was associated with a non-significant increase in risk of death from malignant melanoma in both men and women, and it was the cancer site associated with the highest relative risk of cancer death in women and men combined in the study on height and risk of cause-specific death which included 679 deaths from malignant melanoma [16]. Possible explanations for this association are increased skin surface or higher naevi count in taller individuals, or differences in distribution of other risk factors such as sun exposure [30]. However, adjusting for physical appearance and sun exposure-related factors associated with risk of melanoma did not affect the risk estimates for height and risk in one pooled analysis of 2,083 female cases of malignant melanoma [10].

The incidence of many cancers that occur in both sexes is higher in men than in women. In our study, this was true for colon and rectal cancer and malignant melanoma (data not shown), even after adjustment for smoking status and BMI. Interestingly, one recent study that included 3,466 cases of cancer at shared sites as well as information on several medical and lifestyle risk factors reported that over 30 % of this difference in total cancer risk could be attributed to differences in height [31].

Adult height is determined to a large part by genetic factors, but also by nutrition during childhood and adolescence, mediated by factors in the growth hormone/insulin-like growth factor axis, and other factors such as living conditions and serious disease in childhood [32, 33]. Circulating levels of IGF-1 in adulthood are associated with height in men and inconsistently also in women [34, 35] and IGF-1 has consistently been associated with increased risk of cancer of the breast, prostate, and colorectum [35–38].

There are genes that are related both to increased height and to oncogenic pathways such as c-Myc, p53, and SMAD3, which are related to increased risk of cancer tumorigenesis [39]. Also, height-related SNPs have been associated with risk of testicular cancer [40]. The association between height and cancer might also be influenced by differences in other risk factors for cancer such as smoking and obesity across the height categories. However, in previous studies, tall subjects tended to smoke less and to have lower BMI [2, 13], two factors that are associated with decreased risk of cancer. In our study, tall subjects had lower BMI, whereas tall women smoked more and tall men smoked less than shorter individuals. Since there were no significant interactions between height and smoking status and risk of cancer in women, we do not believe that the slightly higher proportion of smokers in tall women can explain their increased risk of cancer.

Our study is, to the best of our knowledge, the first to analyze height in relation to incidence and death of all-sites cancer in the same cohort. For cancer with short overall survival, the estimates of incidence and mortality are expected to concur. In our study, this is mainly evident for the inverse association between height and risk of diagnosis and death of gastric cancer in men. For cancers with longer overall survival, such as cancer of the breast and colon in women in our study, the association between height and risk of cancer death might also to some part indicate an association to more aggressive disease at diagnosis or to progression. However, mortality in cancer is strongly influenced by stage of disease at diagnosis and treatment, factors that we were unable to control for in the present study.

## Conclusion

Our data from a large prospective pooled European cohort study provide further evidence that height is associated with increased risk of cancer and cancer death, indicating that factors related to height such as genetic and hormonal factors stimulate both cancer development and progression.

## Abbreviations

Me-Can: Metabolic syndrome and cancer project
Oslo: Oslo study I cohort
NCS: Norwegian counties study
CONOR: Cohort of Norway
40-y: Age 40-programme
VHM&PP: Vorarlberg health monitoring and prevention programme
VIP: Västerbotten intervention project
MPP: Malmö preventive project
BMI: Body mass index
SD: Standard deviation
vs.: Versus
ICD: International statistical classification of diseases
Cm: Centimeters
